# Sputter-Deposited Amorphous Li_3_PO_4_ Solid Electrolyte Films

**DOI:** 10.1021/acsomega.2c02104

**Published:** 2022-06-08

**Authors:** Tsuyoshi Ohnishi, Kazunori Takada

**Affiliations:** Center for Green Research on Energy and Environmental Materials, National Institute for Materials Science (NIMS), 1-1 Namiki, Tsukuba, Ibaraki 305-0044, Japan

## Abstract

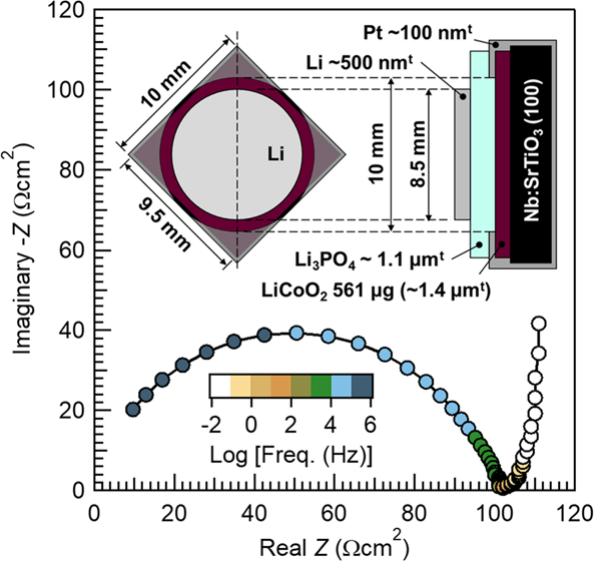

This paper reports the thin-film synthesis of Li_3_PO_4_ solid electrolytes by RF magnetron sputtering. A relatively high ionic conductivity of more than 1 × 10^–6^ S cm^–1^ is achieved. It is revealed that the crystallization of Li_3_PO_4_ impedes ionic conduction, and a moderate amount of O_2_ addition to Ar suppresses the crystallization and guarantees long-term deposition. Another important finding in this study is that when Li_3_PO_4_ is deposited on a LiCoO_2_ film to construct a thin-film battery, the LiCoO_2_ film can be damaged depending on the substrate bias potential relative to the cathode potential propagated through the sputtering plasma. Active control of the bias potential to avoid the damage realizes negligible interface resistance in the thin-film battery.

## Introduction

Solid-state Li-ion batteries are promising next-generation power supplies to replace current Li-ion batteries because of their superior features such as high energy density, long cycle life, and safety. However, it is reported that the interface between a cathode material and a solid electrolyte in a solid-state battery can be rate-determining and thus governs the power density.^1^ Converting solid-state batteries into the thin-film form is an effective way to investigate the interface properties since it simplifies the geometry and provides important information about the interfaces.^2−5^

Lithium phosphorus oxynitride (LiPON) is widely used as a solid electrolyte layer in thin-film batteries because of its relatively high ionic conductivity (∼3 × 10^–6^ S cm^–1^). LiPON was first developed by Bates et al.^6^ by sputtering a Li_3_PO_4_ target in pure N_2_. Their Li/LiPON/LiCoO_2_ thin-film batteries operate for more than 30,000 cycles with a capacity fading of less than 5%.^2^ Since then, a number of thin-film batteries with LiPON have been reported.^3,7,8^ However, recent first-principles calculations indicate that LiPON is not thermodynamically stable, but kinetically stabilized, upon contact with Li metal and LiCoO_2_,^9^ and the calculation results are consistent with experimental results.^10,11^ Although partial replacement of O with N (and Li uptake) improves the ionic conductivity, and the conductivity reaches 6.4 × 10^–6^ S cm^–1^ with simultaneous Li enrichment in the target,^12^ the incorporation of N into Li_3_PO_4_ narrows its electrochemical stability window according to the aforementioned calculations.^9^

Li_3_PO_4_ itself is also used as a solid electrolyte layer in thin-film batteries. Bates et al. examined it along with LiPON by sputtering a Li_3_PO_4_ target with 40% O_2_ in Ar. However, the conductivities of their Li_3_PO_4_ films were as low as 7 × 10^–8^ S cm^–1^;^6^ another group also reported similar values,^7^ and in both of these studies, Li_3_PO_4_ films were deposited by radiofrequency (RF) magnetron sputtering. Meanwhile, Li_3_PO_4_ films prepared by pulsed laser deposition (PLD) using a high-photon-energy ArF excimer laser showed a relatively higher ionic conductivity of ∼5 × 10^–7^ S cm^–1^,^13,14^ and thin-film batteries made with the PLD Li_3_PO_4_ operate rather well.^14−16^

Here, we report the Li_3_PO_4_ solid electrolyte film synthesis by RF magnetron sputtering with a much improved ionic conductivity. Although there are difficulties in LiPON synthesis in terms of controlling the amount of N incorporated and the simultaneous Li addition to achieve charge neutrality, Li_3_PO_4_ synthesis is much simpler. We also report thin-film batteries constructed by depositing Li anodes and Li_3_PO_4_ on LiCoO_2_ epitaxial thin films.

## Results and Discussion

**Figure 1:**
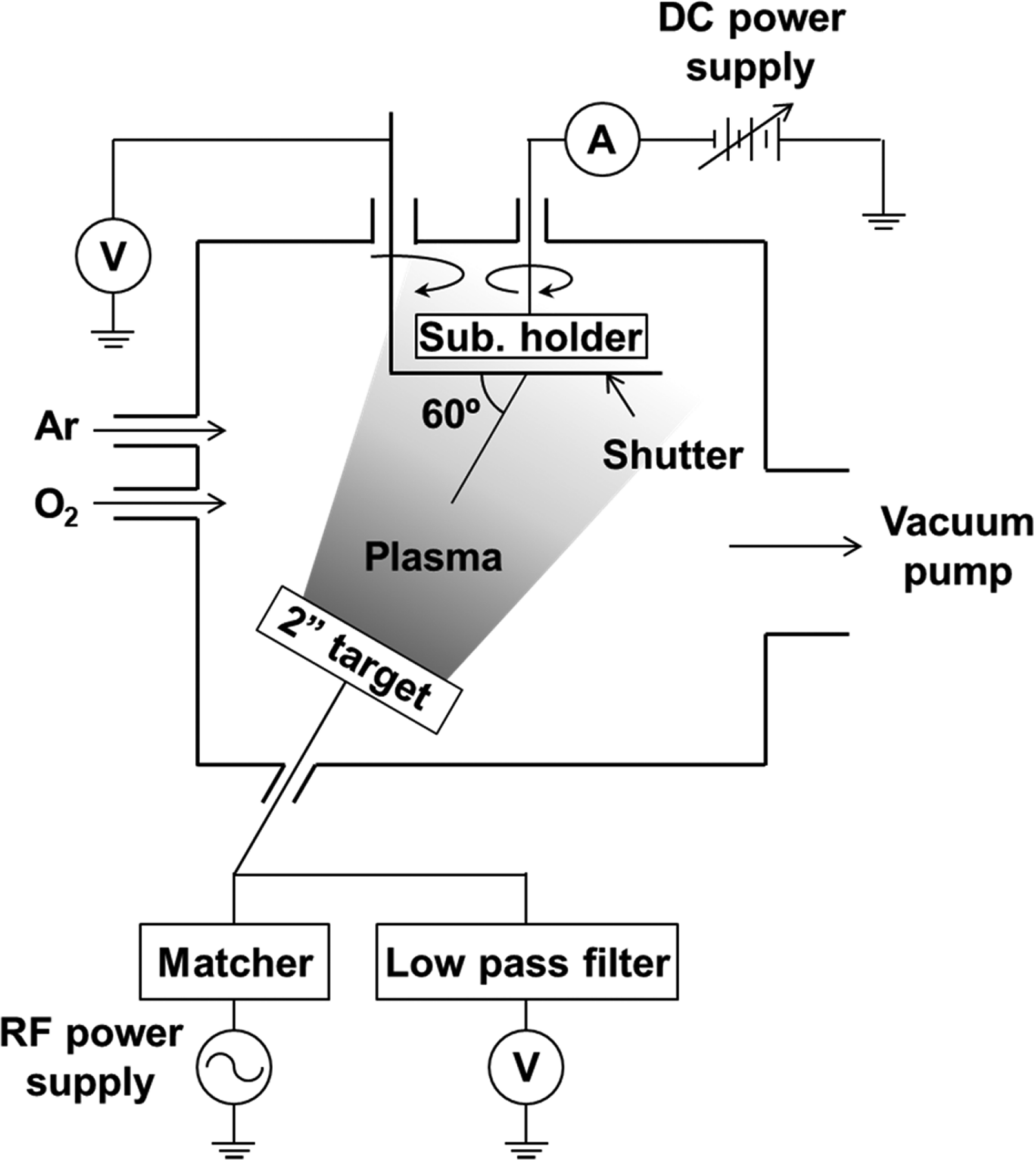
Schematic configuration of a specially designed RF magnetron sputtering system.

A schematic configuration of our specially designed RF magnetron sputtering system is shown in Figure 1. Since multiple sputter cathodes with 2″-diameter targets are equipped, each cathode is oriented to the center of a 2″-diameter substrate holder with a 60° incident angle. The substrate holder is continuously rotated during deposition, and 10 mm square or 10 mm diameter substrates are located around the middle radius position of the 2″ inconel holder for simultaneous multiple deposition. Ar as well as O_2_ gases can be introduced through mass flow controllers. The chamber is evacuated by a turbo molecular pump, and a conductance-controllable gate valve is equipped between the chamber and the pump to adjust the chamber pressure independent of the gas flow rate. The substrate holder potential can be adjusted by a bipolar direct current (DC) power supply. Stainless steel and 0.5 wt % Nb-doped SrTiO_3_ substrates were used, and they were electronically connected to the substrate holder during Li_3_PO_4_ deposition. For the Nb:SrTiO_3_ substrates, a piece of metal Mg was bridged to make a low-resistance connection with the inconel holder. The sputtering plasma potential around the substrate position can be measured by a substrate shutter. Besides, the cathode DC potential can be measured through a low-pass filter during RF sputtering.

### Improvement of Li_3_PO_4_ Conductivity

**Figure 2:**
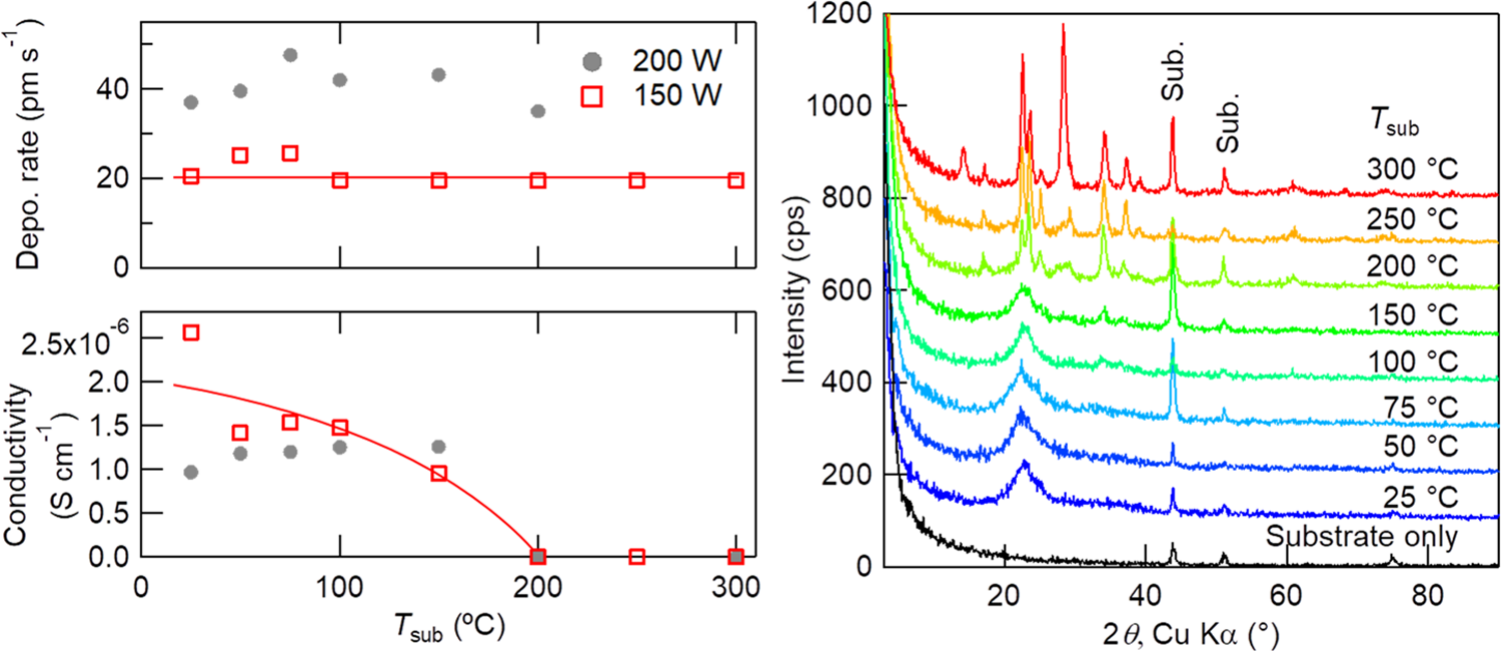
Left panels: *T*_sub_ dependences of the film deposition rate (top) and ionic conductivity (bottom). Lines are visual guides. Right panel: *T*_sub_ dependence of GIXRD patterns of 2 h-deposited Li_3_PO_4_ films on stainless steel substrates with an incident angle of 0.25°. A GIXRD pattern from a substrate without Li_3_PO_4_ deposition is also shown at the bottom. Deposition conditions are as follows: RF power, 150 W; Ar, 20 sccm; O_2_, 5 sccm; total pressure, 0.6 Pa; target–substrate distance, 150 mm; and substrate bias potential, +0.5 V.

It is obvious from Figure 2 that the crystallization of Li_3_PO_4_ film drastically decreases the ionic conductivity. The figures show the substrate temperature (*T*_sub_) dependence of the film deposition rate, ionic conductivity, and X-ray diffraction (XRD) patterns of 2 h-deposited Li_3_PO_4_ films on mirror-polished stainless steel substrates. The 10 mm square and 0.5 mm-thick stainless steel substrate, which works as the bottom blocking electrode, was vacuum-annealed before use to remove the insulative oxidation layer on the surface. The deposition rate was evaluated by the film thickness measured with X-ray reflectance measurement, and ionic conductivity was estimated by alternating-current (AC) impedance measurements with 2 mm diameter Pt blocking electrodes deposited by DC magnetron sputtering. The AC impedance data were obtained in the frequency range of 5 × 10^5^–0.01 Hz with an AC amplitude of 20 mV, and ionic conductivity was estimated from the diameter of the semicircle at a higher-frequency region by fitting. The XRD patterns were measured by the surface-sensitive grazing-incidence method (GIXRD), where the X-ray incident angle is fixed at 0.25° and the intensity is recorded with 2*θ* scanning. Ar and O_2_ gas flow rates were 20 and 5 sccm, respectively, and the total gas pressure was controlled to be 0.6 Pa during deposition. A RF power of 150 W was used, and 200 W data are also plotted in the left panel of Figure 2 for a comparison.

The deposition rate, i.e., the film thickness, is almost constant and independent of *T*_sub_, but the ionic conductivity is sensitive to *T*_sub_; a higher *T*_sub_ results in lower conductivity. According to the GIXRD results, when *T*_sub_ is lower than 150 °C, the Li_3_PO_4_ film is in an amorphous state, showing a halo centered at 2*θ* = 23°. On the other hand, when *T*_sub_ is higher, sharp diffraction peaks appear, which correspond to the Li_3_PO_4_ crystal phase, and at 300 °C, additional peaks at 2*θ* ≈ 14 and 28° appear, which are attributable to the Li_4_P_2_O_7_ crystal phase. Because the *T*_sub_ at the starting of crystallization coincides well with that during the conductivity drop, it is concluded that the crystallization of Li_3_PO_4_ impedes ionic conduction, and an amorphous state is essential for high ionic conductivity. The activation energy estimated from the temperature dependence of ionic conductivity of the amorphous films in the range of 200–350 K was 0.53–0.55 eV. The obtained activation energy and frequency dependence of the impedance were similar to those reported for a PLD-deposited film under an O_2_ atmosphere (0.58 eV),^15^ but quite different from that of a RF-sputtered film deposited under pure Ar (0.38 eV),^7^ suggesting the importance of O_2_ introduction. Although low *T*_sub_ is preferable to make the films amorphous, the substrate is heated up by sputtering plasma during much longer deposition processes, resulting in an unstable *T*_sub_ in our deposition system, because it does not have a substrate cooler. Heating at moderate temperatures between 50 and 150 °C is reliable to keep *T*_sub_ constant throughout the deposition and to deposit amorphous films. Although the 200 W data show higher deposition rates (almost double), the higher cathode power tends to damage the target surface severely and quickly (e.g., by causing cracking and color change); thus, a lower RF power is preferred for long-term deposition.

**Figure 3:**
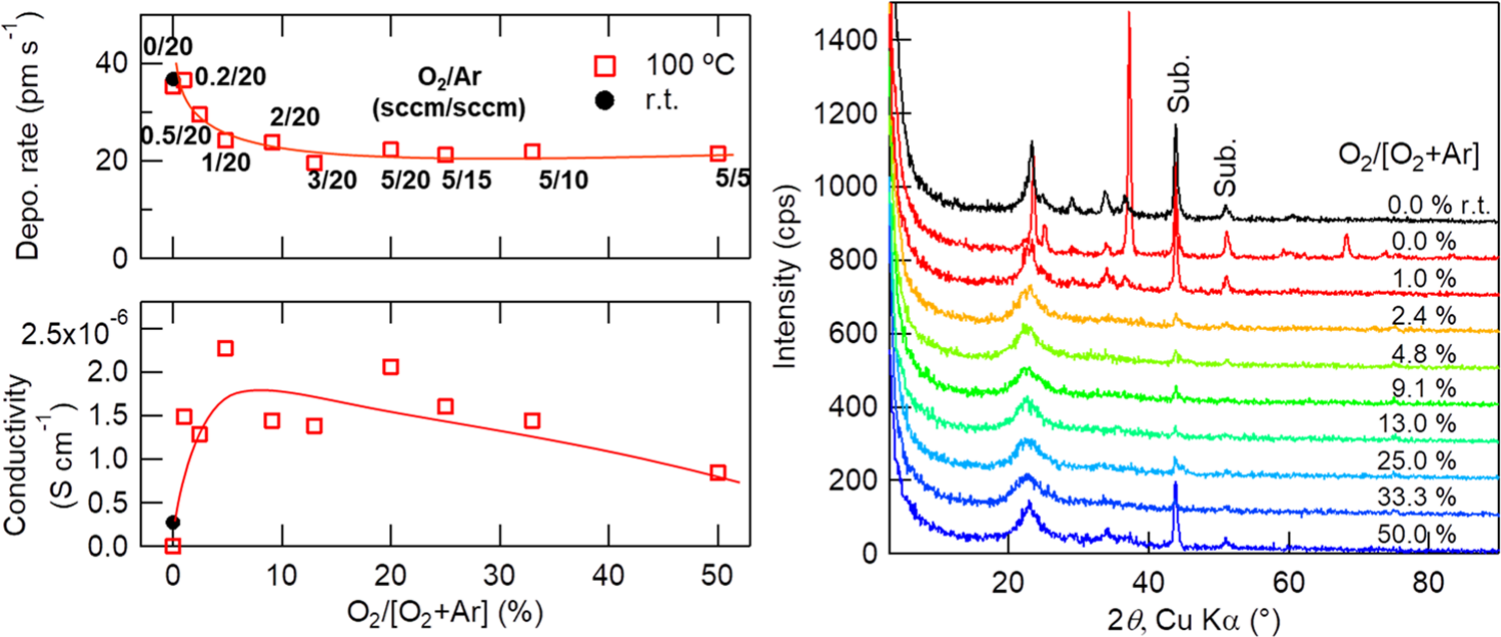
Left panels: O_2_ and Ar gas ratio dependences of the film deposition rate (top) and ionic conductivity (bottom). Lines are visual guides. Right panel: the same dependence of GIXRD patterns of 2 h-deposited Li_3_PO_4_ films on stainless steel substrates with an incident angle of 0.25°. Deposition conditions are as follows: *T*_sub_, 100 °C; RF power, 150 W; total pressure, 0.6 Pa; target–substrate distance, 150 mm; and substrate bias potential, +0.5 V.

Figure 3 shows the *T*_sub_ dependence of the mixing ratio of O_2_ and Ar gases, in the same manner as in Figure 2, under a total pressure of 0.6 Pa, which was controlled by the conductance valve. According to the results of Figure 2, a *T*_sub_ of 100 °C is selected, and the room-temperature deposition is also examined without O_2_ introduction. It is obvious that the deposition rate is higher when none or a small amount of O_2_ is introduced. However, the ionic conductivity is low (less than 10^–6^ S cm^–1^) when no O_2_ is introduced. GIXRD results indicate that Li_3_PO_4_ is crystallized clearly when the O_2_ ratio is 1% or less and only slightly when it is 50%. The latter conditions seem similar to those examined by Bates et al. reporting low conductivity;^6^ thus, it can be concluded that O_2_ is necessary to avoid crystallization; however, excess of O_2_ also results in crystallization and decreases the conductivity. In addition, a thin film deposited at room temperature reveals the importance of O_2_ introduction. The Bragg peaks indicating crystallization are observed for the thin film deposited without O_2_ introduction, even though the film is deposited without substrate heating, and the film shows low ionic conductivity. It means that O_2_ gas is anyway needed to suppress the crystallization of Li_3_PO_4_. Because sputtering is a vacuum process and the deposited film is an oxide, the film tends to become oxygen-deficient. In the field of thin-film growth of high *T*_c_ superconducting and other functional oxides, it is well-known that the melting point (*T*_melt_) and crystallization temperature of oxide materials tend to be lowered when oxygen is deficient, and thus, high-crystallinity thin films of oxide materials with high *T*_melt_ can be obtained via a vacuum process,^17^ e.g., molecular beam epitaxy and PLD, under much lower *T*_sub_ relative to their *T*_melt_ values. Besides, oxygen deficiency is introduced not only in the deposited films but also in the sputtering target, resulting in serious target damage. O_2_ gas introduction is therefore necessary to suppress film crystallization and to avoid target damage in the long-term deposition for a thick solid electrolyte layer. O_2_ introduction also makes the deposited films stable in air; otherwise, the transparent films devitrify after long-term storage in air, probably because of the humidity.

**Figure 4:**
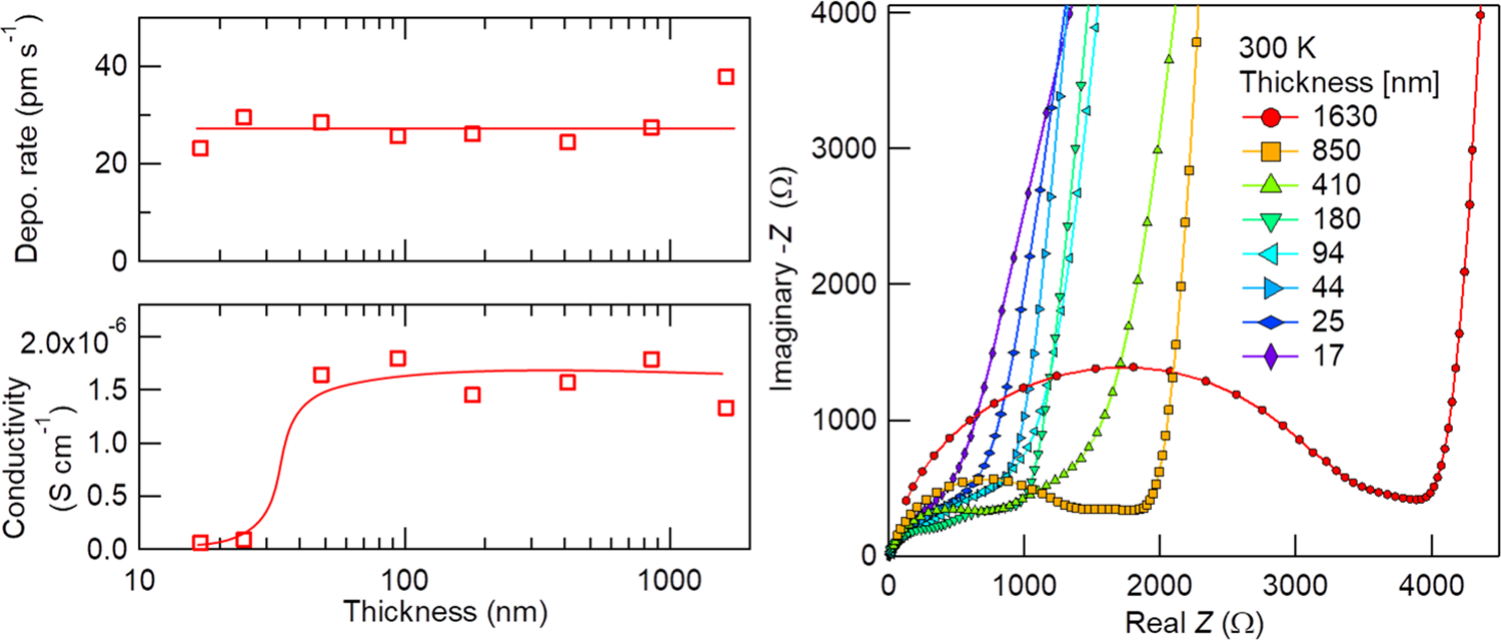
Left panels: thickness dependences of the film deposition rate (top) and ionic conductivity (bottom). Lines are visual guides. Right panel: deposited thickness dependence of AC impedance Nyquist plots. Deposition conditions were as follows: *T*_sub_, 100 °C; RF power, 150 W; Ar, 20 sccm; O_2_, 1 sccm; total pressure, 0.6 Pa; target–substrate distance, 150 mm; and substrate bias potential, +0.5 V. The AC impedance data were taken in the frequency range of 5 × 10^5^–0.01 Hz with an AC amplitude of 20 mV.

Figure 4 shows the film thickness dependence of the deposition rate, ionic conductivity, and AC impedance Nyquist plots. Deposition conditions are as follows: *T*_sub_, 100 °C; RF power, 150 W; Ar, 20 sccm; O_2_, 1 sccm; and total pressure, 0.6 Pa. When the thickness fringes were unclear in X-ray reflectance curves for films thicker than the typical 200 nm, the thickness was evaluated with a stylus profilometer, i.e., measuring the step height made by peeling off masking tapes. It appears that films with a thickness of 50 nm or more are needed to guarantee ionic conduction because all six Pt pads prepared on a 44 nm or thicker Li_3_PO_4_ film were electronically open and showed similar conductivities, but on a 25 nm or thinner Li_3_PO_4_ film, the conductivity was low and some Pt pads were short-circuited already. The constant deposition rate and ionic conductivity observed in the thickness range beyond 50 nm demonstrate that long-term and stable deposition is possible under the current conditions. For the preceding experiments, including this thickness dependence, the substrate potential was kept at +0.5 V by the DC power supply, and the target–substrate distance was fixed to be 150 mm during deposition.

### Damage to the Underlying LiCoO_2_ Film in Battery Devices

**Figure 5:**
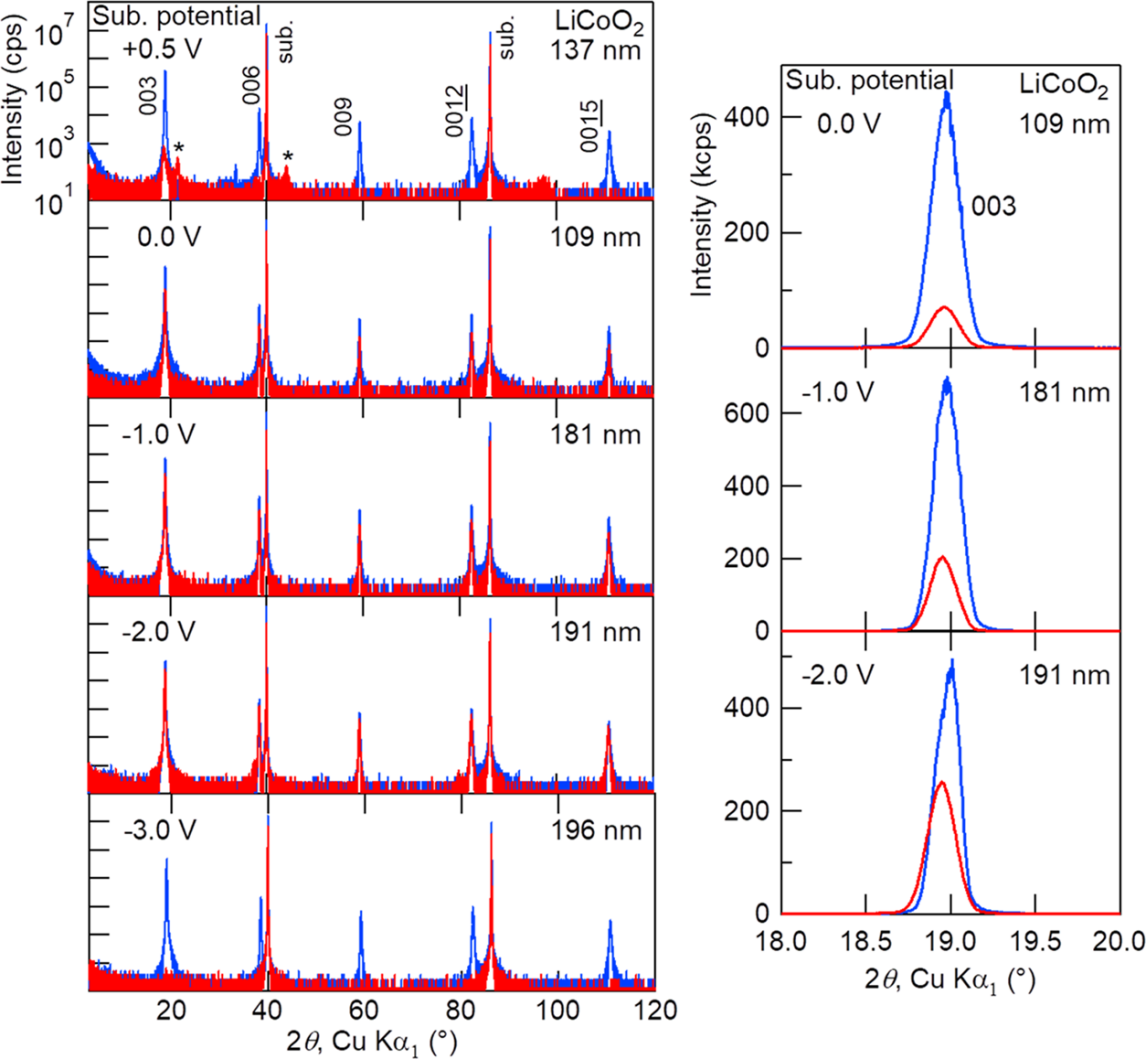
Red curves: 2*θ*–*ω* scan XRD patterns of 15 h Li_3_PO_4_-deposited (2–2.5 μm thick) LiCoO_2_ thin films under different substrate bias potentials during sputtering. Deposition conditions were as follows: *T*_sub_, 100 °C; RF power, 120 W; Ar, 20 sccm; O_2_, 7 sccm; total pressure, 0.6 Pa; and target–substrate distance, 150 mm. XRD patterns before Li_3_PO_4_ deposition are also shown in blue curves, and the LiCoO_2_ thickness is given on the right top of each panel. Right panels are magnified views around LiCoO_2_ 003 in a linear intensity scale at potentials of 0.0, −1.0, and −2.0 V. Diffraction peaks marked by “*” in the left top panel indicate the O1 phase 00*l*.

Li_3_PO_4_ films were deposited on PLD-grown epitaxial LiCoO_2_ thin films. The substrates were 0.5 wt % Nb-doped SrTiO_3_ (111) single crystals with a 10 mm square or 10 mm diameter and 0.5 mm thickness, and LiCoO_2_ grew in the *c*-axis orientation with a thickness of 100–200 nm. Details of the LiCoO_2_ thin-film synthesis are described elsewhere.^18,19^Figure 5 shows the 2*θ*–*ω* scan XRD patterns of 15 h Li_3_PO_4_-deposited (2–2.5 μm thick) LiCoO_2_ thin films under different substrate bias potentials during sputtering. Diffraction patterns before Li_3_PO_4_ deposition are also shown in blue curves. Deposition conditions were as follows: *T*_sub_, 100 °C; RF power, 120 W; Ar, 20 sccm; O_2_, 7 sccm; and total pressure, 0.6 Pa. Because the deposition rate of Li_3_PO_4_ was stable under certain conditions, the deposited thickness was controlled by the deposition time. It is obvious that there is a clear substrate bias potential dependence of LiCoO_2_ crystallinity after Li_3_PO_4_ deposition. When the potential is lower than −3 V or higher than +0.5 V, LiCoO_2_ diffraction peaks disappear or the intensity decreases drastically, i.e., the LiCoO_2_ crystal lattice is destroyed. Besides, it seems that there is an optimal substrate potential, and −2.0 V is close to the optimal value in these depositions as the intensity decrease of LiCoO_2_ diffraction is minimal. It should be noted here that the conductivity of Li_3_PO_4_ films deposited on stainless steel substrates within this potential range is almost constant (≈1.2 × 10^–6^ S cm^–1^), independent of the substrate bias potential.

**Figure 6:**
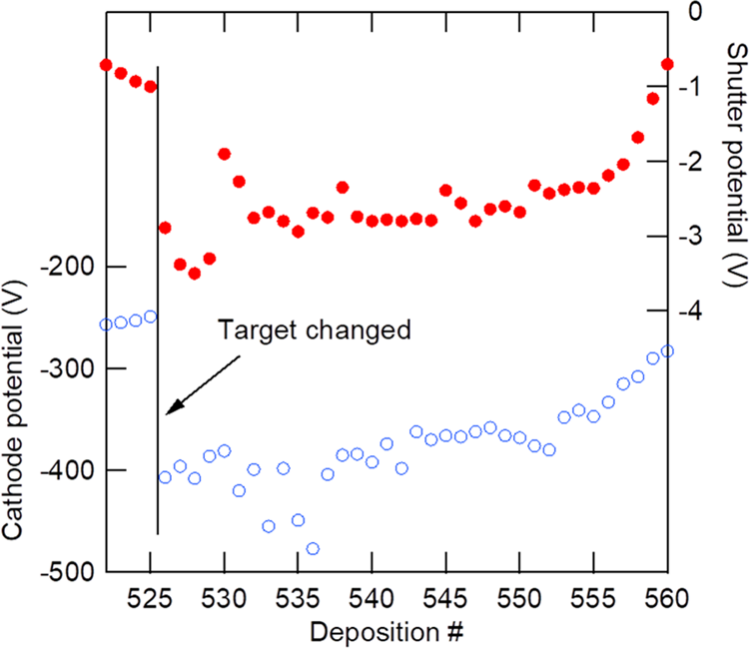
Top: shutter potential; bottom: cathode potential; RF power was set at 100 W before each 5 h deposition. Deposition conditions were as follows: Ar, 20 sccm; O_2_, 10 sccm; total pressure, 0.8 or 1.0 Pa; and target–substrate distance: 95 mm. The target was changed between depositions #525 and #526.

It appears that an optimal substrate bias potential exists; however, it is not constant but varies gradually with each deposition. Figure 6 shows a plot of the cathode potential when the RF power is set at 100 W before each 5 h deposition. Other deposition conditions were as follows: Ar, 20 sccm; O_2_, 10 sccm; total pressure, 0.8 or 1.0 Pa; and the target–substrate distance was shortened to 95 mm from and after this experiment to approximately double the deposition rate. The target was changed between depositions #525 and #526. The target change alters the various deposition conditions, but the biggest change is in the DC cathode potential during RF sputtering. When the target is being worn out, the cathode potential increases and reaches around −250 V under these conditions, and finally, the rear indium bond and Cu backing plate appear around the eroding part of the Li_3_PO_4_ target. In the case of a new target, the cathode potential is as low as around −400 V. Scattering of the cathode potential is mainly caused by the total pressure change. It should also be noted that the total pressure is related to the deposition rate as well as the shutter potential. When the pressure is increased in the range of 0.6–1.0 Pa, the deposition rate slightly decreases mainly due to stronger gas scattering, and the shutter potential increases and approaches 0 V. It should be noted that the cathode potential has a strong correlation to the shutter potential around the substrate position as shown in Figure 6 (top). Although the shutter potential is always negative, it changes and correlates to the cathode potential. It can be assumed that the optimal substrate bias potential that preserves the LiCoO_2_ crystal quality after the Li_3_PO_4_ deposition is related to the cathode potential, and it changes gradually with the change in the target surface state.

**Figure 7:**
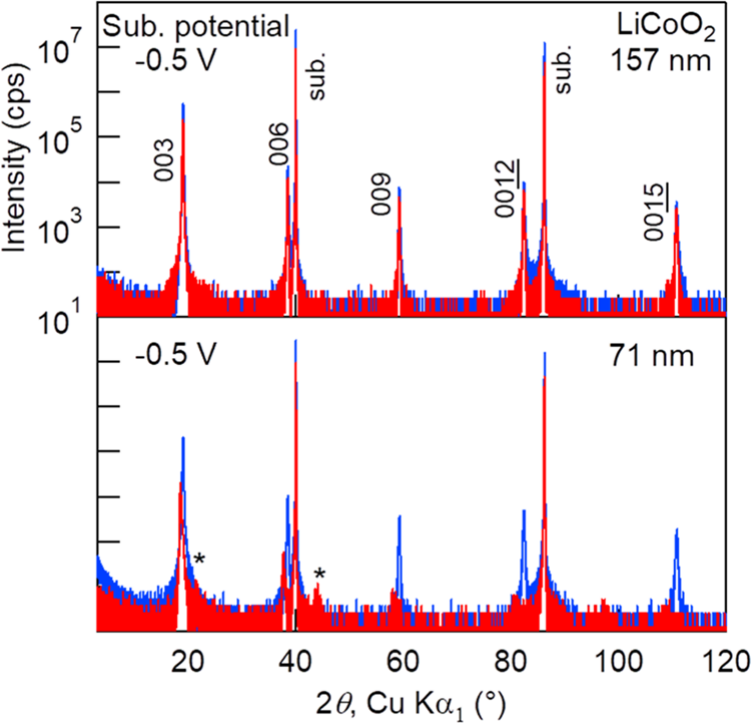
Red curves: 2*θ*–*ω* scan XRD patterns of two 15 h Li_3_PO_4_-deposited LiCoO_2_ thin films under a substrate bias potential of −0.5 V. Other deposition conditions were as follows: *T*_sub_, 100 °C; RF power, 100 W; Ar, 20 sccm; O_2_, 10 sccm; total pressure, 0.6 Pa; and target-substrate distance: 95 mm. XRD patterns before Li_3_PO_4_ deposition are also shown in blue curves; the LiCoO_2_ thickness is given on the right top of each panel. Diffraction peaks marked by “*” in the bottom panel indicate the O1 phase 00*l*.

The change in the optimum bias potential can be seen in Figure 7. It shows 2*θ*–*ω* scan XRD patterns of two 15 h Li_3_PO_4_-deposited LiCoO_2_ thin films under the same substrate bias potential of −0.5 V, but before and after the target change. Other deposition conditions were as follows: *T*_sub_, 100 °C; RF power, 100 W; Ar, 20 sccm; O_2_, 10 sccm; and total pressure, 0.6 Pa. Between the two depositions, the Li_3_PO_4_ target was changed for a new one because the target was worn out. The LiCoO_2_ crystallinity of the top one is almost preserved, whereas that of the bottom one is degraded severely. It means that the optimal substrate bias potential was around −0.5 V, but it has shifted after the target change. Thin-film batteries made by ∼500 nm-thick Li anode deposition with vacuum thermal evaporation on Li_3_PO_4_ films support the tendency; an open-circuit voltage (ocv) of the thin-film battery with the top sample just after cell construction was 3.9 V, whereas that of the bottom one was 4.3 V.

Here, we discuss what happens when the substrate bias potential is not optimal. Because Li_3_PO_4_ and LiCoO_2_ do not react with each other at substrate temperatures as low as 100 °C, there must be other reasons that relate to the potential. In a battery, the LiCoO_2_ cathode can be damaged by overcharging and over-discharging, with too high and too low cutoff voltages, respectively. In the sputtering process, the cathode DC potential always becomes negative to sputter the target material by positively ionized Ar gases. Even though it depends on the total pressure, the target–substrate distance, and the on-axis/off-axis geometry,^7^ the target and substrate are connected to each other by plasma, which is an electron-conductive gas; thus, the substrate holder is subjected to the cathode potential to some extent, as shown in Figure 6. Since LiCoO_2_ is underneath the previously deposited Li_3_PO_4_, which is connected to the plasma, LiCoO_2_ can be charged or discharged depending on the substrate bias potential relative to the plasma potential. In Figure 7 (bottom), the LiCoO_2_ film appears to be overcharged as the X-ray diffraction intensity is considerably decreased, and additional reflections appearing at 2θ ≈ 21.6 and 44.0° are attributable to the 001 and 002 diffractions of the O1 phase, respectively, which is an overcharged phase of LiCoO_2_.^20,21^ Besides, the ocv of the as-constructed battery was 4.3 V, which is higher than the standard charging cutoff voltage of 4.2 V. In Figure 5, the top film is also overcharged, but the bottom one seems over-discharged as LiCoO_2_ cannot take up extra Li,^22^ unlike the LiNi_0.5_Mn_1.5_O_4_ cathode,^16,23^ and thus the LiCoO_2_ crystal collapses.

In the literature, disordered LiCoO_2_^24^ and Li_2_MnO_4_^25^ cathode layers are observed at the interface with LiPON in cross-sectional images obtained by a scanning transmission electron microscope. Although LiPON is deposited by sputtering the Li_3_PO_4_ target with pure N_2_ instead of an Ar and O_2_ mixture, as in our study, effects of the plasma potential arise likewise: if the substrate potential is not adequate, the cathode layer can be damaged from the LiPON interface. Related to the latter Li_2_MnO_4_, the same authors report Li_*x*_MnO_2_ cathode formation from a MnO_2–*x*_ layer by depositing a LiPON layer on top of it.^26^ They claim that the LiPON deposition infuses Li ions into MnO_2–*x*_. Considering the Li_2_MnO_4_ disordering and formation of Li_*x*_MnO_2_ from MnO_2–*x*_, when the substrate potential is lower than that of the plasma, discharging (Li-ion infusion) takes place, probably due to the charge build-up of negative plasma by an electronically isolated substrate holder.

The shutter potential can be a good reference to determine the optimal substrate bias potential; however, it is not stable enough for long-term Li_3_PO_4_ sputtering, e.g., 10 h deposition; it can shift due to changes in the target surface state, e.g., sudden target cracking, oxygen deficiency introduction, and so on. Therefore, even if the substrate bias potential is determined once from the shutter potential before the deposition, the shift of the plasma potential deviates the substrate bias potential from the optimum value during deposition, which results in low experimental reproducibility. Here, we introduce a strategy to solve the reproducibility problem rather easily. During Li_3_PO_4_ deposition, the substrate bias potential is adjusted in real time so that the current meter between the substrate holder and DC power supply (Figure 1) shows zero current. Ideally, no current flows when the substrate bias potential and potential induced from the plasma are balanced. With this active control, we successfully fabricated 10 mm-scale Li/Li_3_PO_4_/LiCoO_2_ thin-film batteries exhibiting high performance; some results have been published already,^21,27^ and another example is described below.

**Figure 8:**
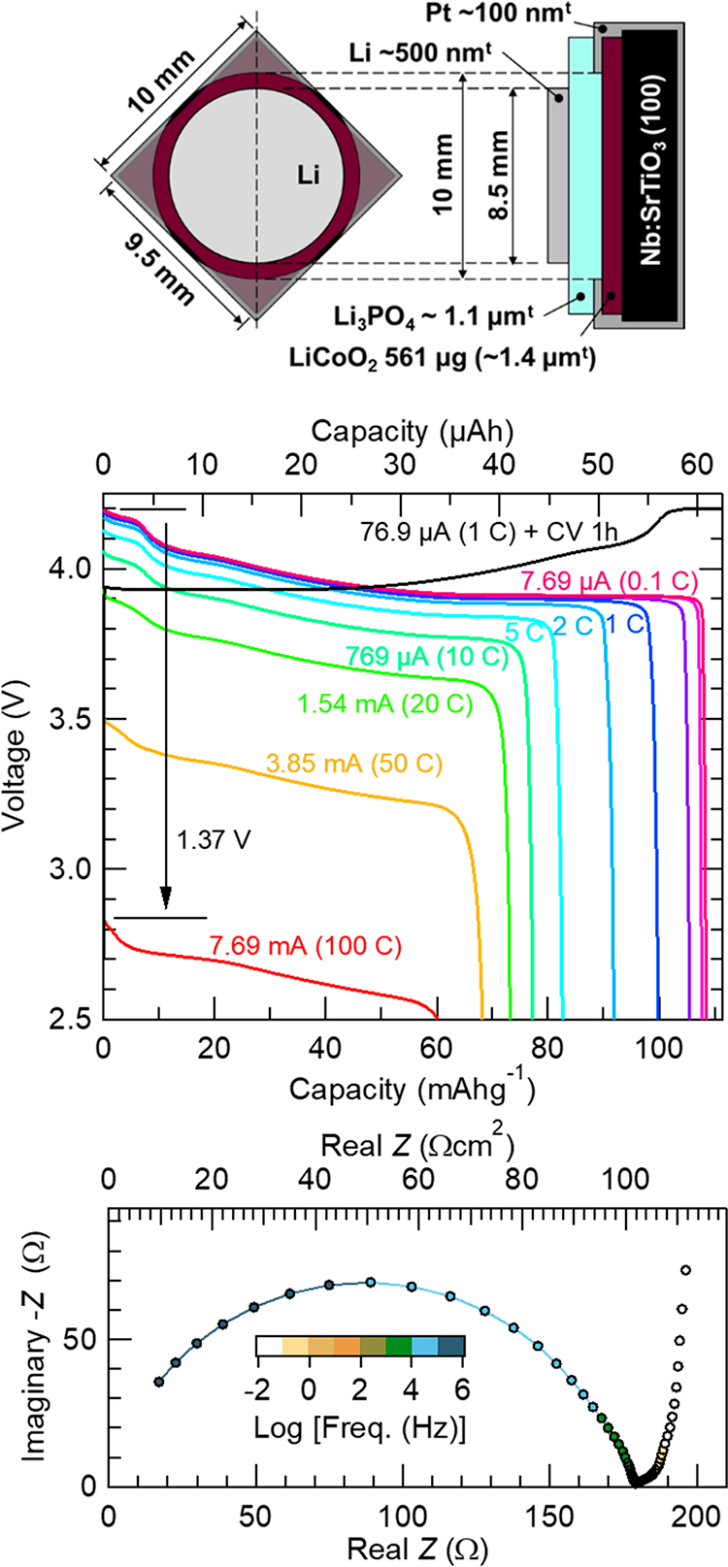
Room-temperature performances of a Li (∼500 nm)/Li_3_PO_4_ (1.1 μm)/LiCoO_2_ (561 μg) thin-film battery. Top: the battery device structure; middle: discharge curves for different C-rates; bottom: AC impedance Nyquist plot at 4.2 V charged state, where the top *x*-axis is areal resistance calculated with an 8.5 mm-diameter Li anode. AC impedance data were obtained in the frequency range of 5 × 10^5^–0.01 Hz, with an AC amplitude of 20 mV. Li_3_PO_4_ deposition conditions were as follows: *T*_sub_, 50 °C; RF power, 150 W; Ar, 20 sccm; O_2_, 10 sccm; total pressure, 0.8 Pa; and target–substrate distance, 95 mm.

Figure 8 shows our thin-film battery structure and room-temperature performances of the battery in which Li_3_PO_4_ (ca. 1.1 μm thick) was deposited under the consideration of the above-described over-charging/discharging processes. Li_3_PO_4_ deposition conditions were as follows: *T*_sub_, 50 °C; RF power, 150 W; Ar, 20 sccm; O_2_, 10 sccm; total pressure, 0.8 Pa; and 5 h deposition. The increased total pressure was to reduce the absolute value of the plasma potential. The RF magnetron sputtering-grown LiCoO_2_ was a (104)-oriented epitaxial film on a 10 mm square, 0.5 mm-thick 0.5 wt % Nb-doped SrTiO_3_ (100) substrate, with a film weight of 561 μg (ca. 1.4 μm thick), which was measured with an electronic balance. Details of LiCoO_2_ sputtering growth are reported elsewhere. Before the Li_3_PO_4_ deposition, an ∼100 nm-thick Pt current collector was deposited on a LiCoO_2_ film by DC magnetron sputtering, which is a 10 mm square film with a circular opening with a diameter of 10 mm,^21^ and connected to the substrate holder electronically during Li_3_PO_4_ deposition. Meanwhile, all of our LiCoO_2_ films were air-exposed after synthesis for all purposes including weighing, XRD measurement, and Pt deposition. After Li_3_PO_4_ deposition, a circular Li anode with a diameter of 8.5 mm was formed above the opening of the Pt current collector.^21^ Although the LiCoO_2_ cathode layer was relatively thick, the battery showed a rather high rate capability. Here, a charging/discharging rate of 1 C is defined as 137 mA g^–1^, which is based on the expected capacity when LiCoO_2_ is charged up to Li_0.5_CoO_2_ at 4.2 V.^28^ The thin-film battery was charged at a 1 C constant current up to a cutoff voltage of 4.2 V, followed by a constant-voltage charging at 4.2 V for 1 h before each discharge to guarantee a fully charged state. The capacity of low-rate discharge with 7.69 μA (0.1 C) was 108 mAh g^–1^, whereas the capacity of high-rate discharge with 7.69 mA (100 C) was 60 mAh g^–1^, maintaining >55% of the low-rate discharge. Figure 8 (bottom) shows a Nyquist plot at the 4.2 V charged state. The semicircle at the higher-frequency region originates from the resistance of Li_3_PO_4_, and its ionic conductivity calculated from the *x*-intercept of the semicircle at a lower frequency is 1.1 × 10^–6^ S cm^–1^. It should be noted that other resistances, e.g., the interface resistance between the Li anode and Li_3_PO_4_, or Li_3_PO_4_ and the LiCoO_2_ cathode, are not clearly observed, which is different from previously reported results.^7,14^ Most recently, the reduced interface resistance was reported to be 10.3 Ω cm^2^ between LiCoO_2_ and RF-sputtered Li_3_PO_4_,^29^ and also a further reduced resistance of 8.6 Ω cm^2^ was reported between LiCoO_2_ and LiPON.^7^ As the active area of our battery was 0.567 cm^2^ (8.5 mm-diameter Li anode), at least 15 Ω of interface resistance should appear in the Nyquist plots, if such a resistance exists; however, it cannot be seen in Figure 8 (bottom). The *IR* drop at 7.69 mA discharge is 1.37 V (Figure 8 middle) and thus *R* becomes 178 Ω. The resistance of Li_3_PO_4_ in Figure 8 (bottom) is 179 Ω, which is almost the same value, i.e., no additional resistance exists, even though the LiCoO_2_ film is air-exposed. It strongly suggests that the intrinsic interface resistance of Li/Li_3_PO_4_ or Li_3_PO_4_/LiCoO_2_ is much smaller than that reported and is almost negligible, and this can be achieved by the active control of the substrate potential during Li_3_PO_4_ deposition. In other words, sputtering^7^ or bombardment of high-energy ablated species^14^ during the Li_3_PO_4_ deposition has been proposed to cause damages to the LiCoO_2_ layer, which results in a large interface resistance, and eliminating these causes lowered the interface resistance to ca. 10 Ω cm^2^. However, the interface resistance has not reached the intrinsic value yet due to remaining damages under uncontrolled bias potential. In fact, we also observed such an interfacial resistance (≈10 Ω cm^2^) when the bias potential control was not adequate, and the details are reported in a separate paper.

## Conclusions

This study revealed that RF magnetron sputtering-deposited Li_3_PO_4_ can have a relatively high ionic conductivity of more than 1 × 10^–6^ S cm^–1^, close to that of LiPON, by avoiding Li_3_PO_4_ crystallization; the addition of a certain amount of O_2_ gas into Ar as well as a low substrate temperature are effective to suppress the crystallization. Besides, this study points out the importance of substrate potential control during Li_3_PO_4_ deposition on LiCoO_2_ films, which has been a hidden, but predominant, process parameter for achieving high performance in thin-film batteries.

## Abbreviations

LiPON: lithium phosphorus oxynitride
RF: radiofrequency
PLD: pulsed-laser deposition
*T*_sub_: substrate temperature
XRD: X-ray diffraction
AC: alternating current
GIXRD: grazing-incidence X-ray diffraction
*T*_melt_: melting point
ocv: open circuit voltage

## References

[ref1] Ohta N.; Takada K.; Zhang L.; Ma R.; Osada M.; Sasaki T. Enhancement of the High-Rate Capability of Solid-State Lithium Batteries by Nanoscale Interfacial Modification. Adv. Mater. 2006, 18, 2226–2229. 10.1002/adma.200502604.

[ref2] Wang B.; Bates J. B.; Hart F. X.; Sales B. C.; Zuhr R. A.; Robertson J. D. Characterization of Thin-Film Rechargeable Lithium Batteries with Lithium Cobalt Oxide Cathodes. J. Electrochem. Soc. 1996, 143, 3203–3213. 10.1149/1.1837188.

[ref3] Iriyama Y.; Nishimoto K.; Yada C.; Abe T.; Ogumi Z.; Kikuchi K. Charge-Transfer Reaction at the Lithium Phosphorus Oxynitride Glass Electrolyte/Lithium Manganese Oxide Thin-Film Interface and Its Stability on Cycling. J. Electrochem. Soc. 2006, 153, A821–A825. 10.1149/1.2178647.

[ref4] Yamamoto K.; Iriyama Y.; Asaka T.; Hirayama T.; Fujita H.; Fisher C.A.J..; Nonaka K.; Sugita Y.; Ogumi Z. Dynamic Visualization of the Electric Potential in an All-Solid-State Rechargeable Lithium Battery. Angew. Chem., Int. Ed. 2010, 49, 4414–4417. 10.1002/anie.200907319. 20468017

[ref5] Song J.; Jacke S.; Cherkashinin G.; Schmid S.; Dong Q.; Hausbrand R.; Jaegermann W. Valence Band Offsets of LiPON/LiCoO_2_ Hetero-Interfaces Determined by X-ray Photoelectron Spectroscopy. Electrochem. Solid-State Lett. 2011, 14, A189–A191. 10.1149/2.006112esl.

[ref6] Bates J. B.; Dudney N. J.; Gruzalski G. R.; Zuhr R. A.; A Choudhury A.; Luck C. F.; Robertson J. D. Fabrication and characterization of amorphous lithium electrolyte thin films and rechargeable thin-film batteries. J. Power Sources 1993, 43, 103–110. 10.1016/0378-7753(93)80106-Y.

[ref7] Haruta M.; Shiraki S.; Suzuki T.; Kumatani A.; Ohsawa T.; Takagi Y.; Shimizu R.; Hitosugi T. Negligible “Negative Space-Charge Layer Effects” at Oxide-Electrolyte/Electrode Interfaces of Thin-Film Batteries. Nano Lett. 2015, 15, 1498–1502. 10.1021/nl5035896. 25710500

[ref8] Bates J. B.; Dudney N. J.; Neudecker B.; Ueda A.; Evans C. D. Thin-film lithium and lithium-ion batteries. Solid State Ionics 2000, 135, 33–45. 10.1016/S0167-2738(00)00327-1.

[ref9] Zhu Y.; He X.; Mo Y. Origin of Outstanding Stability in the Lithium Solid Electrolyte Materials: Insights from Thermodynamic Analyses Based on First-Principles Calculations. ACS Appl. Mater. Interfaces 2015, 7, 23685–23693. 10.1021/acsami.5b07517. 26440586

[ref10] Schwöbel A.; Hausbrand R.; Jaegermann W. Interface Reactions between LiPON and Lithium Studied by in-Situ X-Ray Photoemission. Solid State Ionics 2015, 273, 51–54. 10.1016/j.ssi.2014.10.017.

[ref11] Yu X.; Bates J. B.; Jellison G. E.; Hart F. X. A Stable Thin-Film Lithium Electrolyte: Lithium Phosphorus Oxynitride. J. Electrochem. Soc. 1997, 144, 524–532. 10.1149/1.1837443.

[ref12] Suzuki N.; Inaba T.; Shiga T. Electrochemical properties of LiPON films made from a mixed powder target of Li_3_PO_4_ and Li_2_O. Thin Solid Films 2012, 520, 1821–1825. 10.1016/j.tsf.2011.08.107.

[ref13] Kuwata N.; Iwagami N.; Kawamura ArF excimer laser deposition of wide-band gap solid electrolytes for thin film batteries. Solid State Ionics 2009, 180, 644–648. 10.1016/j.ssi.2008.09.010.

[ref14] Shiraki S.; Shirasawa T.; Suzuki T.; Kawasoko H.; Shimizu R.; Hitosugi T. Atomically Well-Ordered Structure at Solid Electrolyte and Electrode Interface Reduces the Interfacial Resistance. ACS Appl. Mater. Interfaces 2018, 10, 41732–41737. 10.1021/acsami.8b08926. 30465729

[ref15] Kuwata N.; Iwagami N.; Tanji Y.; Matsuda Y.; Kawamura J. Characterization of thin-film lithium batteries with stable thin-film Li_3_PO_4_ solid electrolytes fabricated by ArF excimer laser deposition. J. Electrochem. Soc. 2010, 157, A521–A527. 10.1149/1.3306339.

[ref16] Kawasoko H.; Shirasawa T.; Nishio K.; Shimizu R.; Shiraki S.; Hitosugi T. Clean Solid-Electrolyte/Electrode Interfaces Double the Capacity of Solid-State Lithium Batteries. ACS Appl. Mater. Interfaces 2021, 13, 5861–5865. 10.1021/acsami.0c21586. 33494591

[ref17] Yun K. S.; Choi B. D.; Matsumoto Y.; Song J. H.; Kanda N.; Ito T.; Kawasaki M.; Koinuma H.; et al. Vapor-liquid-solid tri-phase pulsed-laser epitaxy of RBa_2_Cu_3_O_7-y_ single-crystal films. Appl. Phys. Lett. 2002, 80, 61–63. 10.1063/1.1432111.

[ref18] Okada K.; Ohnishi T.; Mitsuishi K.; Takada K. Epitaxial growth of LiCoO_2_ thin films with (001) orientation. AIP Adv. 2017, 7, 115011 10.1063/1.4999833.

[ref19] Nishio K.; Ohnishi T.; Akatsuka K.; Takada K. Crystal orientation of epitaxial LiCoO_2_ films grown on SrTiO_3_ substrates. J. Power Sources 2014, 247, 687–691. 10.1016/j.jpowsour.2013.08.132.

[ref20] Amatucci G. G.; Tarascon J. M.; Klein L. C. CoO_2_, the end member of the Li_x_CoO_2_ solid solution. J. Electrochem. Soc. 1996, 143, 1114–1123. 10.1149/1.1836594.

[ref21] Ohnishi T.; Mitsuishi K.; Takada K. In Situ X-ray Diffraction of LiCoO_2_ in Thin-Film Batteries under High-Voltage Charging. ACS Appl. Energy Mater. 2021, 4, 14372–14379. 10.1021/acsaem.1c03046.

[ref22] Godshall N. A.; Raistrick I. D.; Huggins R. A. Relationships among Electrochemical, Thermodynamic, and Oxygen Potential Quantities in Lithium-Transition Metal-Oxygen Molten Salt Cells. J. Electrochem. Soc. 1984, 131, 543–549. 10.1149/1.2115624.

[ref23] Kawasoko H.; Shiraki S.; Suzuki T.; Shimizu R.; Hitosugi T. Extremely low resistance of Li_3_PO_4_ electrolyte/Li(Ni_0.5_Mn_1.5_)O_4_ electrode interfaces. ACS Appl. Mater. Interfaces 2018, 10, 27498–27502. 10.1021/acsami.8b08506. 29989389

[ref24] Wang Z.; Santhanagopalan D.; Zhang W.; Wang F.; Xin H. L.; He K.; Li J.; Dudney N.; Meng Y. S. In Situ STEM-EELS Observation of Nanoscale Interfacial Phenomena in All-Solid-State Batteries. Nano Lett. 2016, 16, 3760–3767. 10.1021/acs.nanolett.6b01119. 27140196

[ref25] Xia Q.; Sun S.; Xu J.; Zan F.; Yue J.; Zhang Q.; Gu L.; Xia H. Self-Standing 3D Cathodes for All-Solid-State Thin Film Lithium Batteries with Improved Interface Kinetics. Small 2018, 14, 1804149 10.1002/smll.201804149. 30467972

[ref26] Xia Q.; Zhang Q.; Sun S.; Hussain F.; Zhang C.; Zhu X.; Meng F.; Liu K.; Geng H.; Xu J.; Zan F.; Wang P.; Gu L.; Xia H. Tunnel Intergrowth Li_x_MnO_2_ Nanosheet Arrays as 3D Cathode for High-Performance All-Solid-State Thin Film Lithium Microbatteries. Adv. Mater. 2021, 33, 2003524 10.1002/adma.202003524. 33336535

[ref27] Kawashima K.; Ohnishi T.; Takada K. High-rate capability of LiCoO_2_ cathodes. ACS Appl. Energy Mater. 2020, 3, 11803–11810. 10.1021/acsaem.0c01973.

[ref28] Ohzuku T.; Ueda A. Solid-State Redox Reactions of LiCoO_2_ (*R*3*m*) for 4 Volt Secondary Lithium Cells. J. Electrochem. Soc. 1994, 141, 2972–2977. 10.1149/1.2059267.

[ref29] Kobayashi S.; Arguelles E. F.; Shirasawa T.; Kasamatsu S.; Shimizu K.; Nishio K.; Watanabe Y.; Kubota Y.; Shimizu R.; Watanabe S.; Hitosugi T. Drastic Reduction of the Solid Electrolyte–Electrode Interface Resistance via Annealing in Battery Form. ACS Appl. Mater. Interfaces 2022, 14, 2703–2710. 10.1021/acsami.1c17945. 34991318

